# Assessment of Systemic Inflammation and Nutritional Indicators in Predicting Recurrence-Free Survival After Surgical Resection of Gastrointestinal Stromal Tumors

**DOI:** 10.3389/fonc.2021.710191

**Published:** 2021-07-26

**Authors:** Zhenhua Lu, Rui Li, Xianglong Cao, Chengyu Liu, Zhen Sun, Xiaolei Shi, Weiwei Shao, Yangyang Zheng, Jinghai Song

**Affiliations:** ^1^ Department of General Surgery, Department of Hepato-bilio-pancreatic Surgery, Beijing Hospital, National Center of Gerontology, Institute of Geriatric Medicine, Chinese Academy of Medical Sciences, Beijing, China; ^2^ The Key Laboratory of Geriatrics, Beijing Institute of Geriatrics, Beijing Hospital, National Center of Gerontology, National Health Commission; Institute of Geriatric Medicine, Chinese Academy of Medical Sciences, Beijing, China; ^3^ 9th Department, Plastic Surgery Hospital, Peking Union Medical College, Chinese Academy of Medical Sciences, Beijing, China; ^4^ Department of General Surgery, Department of Gastrointestinal Surgery, Beijing Hospital, National Center of Gerontology, Institute of Geriatric Medicine, Chinese Academy of Medical Sciences, Beijing, China

**Keywords:** gastrointestinal mesenchymal tumors, systemic inflammation, nutritional index, recurrence-free survival, prognosis analysis

## Abstract

**Background:**

Recent studies have shown that the systemic inflammation and nutritional indicators are prognostic for a variety of malignancies. However, only limited data have so far demonstrated their usefulness in gastrointestinal mesenchymal tumors (GIST).

**Methods:**

We retrospectively analyzed the data of GIST patients who underwent radical surgery in Beijing hospital from October 2004 to July 2018. The area under the receiver operating characteristic curve (AUC) was used to compare several commonly used inflammatory and nutritional indicators. The indicators with largest AUC were further analysis. Optimal cut-off values of those indicators in predicting recurrence-free survival (RFS) were determined. Kaplan-Meier curve and the time-dependent receiver operating characteristic (ROC) curve were used to assess the prognostic values. We then used univariate and multivariate Cox regression analyses to identify prognostic factors that were associated with RFS.

**Results:**

In total, 160 patients who underwent surgery for GIST were included in the study. The median survival time was 34.5 months, with 1-, 3-, and 5-year RFS rates of 96.1%, 84.7%, and 80.8%, respectively. The inflammatory and nutritional indicators with largest AUC were Systemic immunoinflammatory Index (SII) and Geriatric Nutrition Risk Index (GNRI), reached 0.650 and 0.713, respectively. The optimal cutoff of GNRI and SII were 98.3, and 820.0, respectively. Univariate analysis showed that GNRI, SII, KI67, surgery method, tumor location, tumor size, and mitotic index were all significant prognostic indicators of RFS. After multivariate Cox analysis, independent prognostic factors for RFS in GIST included tumor location, mitotic index, tumor size, and GNRI (HR=2.802,95% CI: 1.045 to 7.515, p = 0.041). Besides, SII also tended to be associated with RFS (HR = 2.970, 95% CI: 0.946 to 9.326, p = 0.062).

**Conclusions:**

High GNRI is an independent prognostic factor for RFS in GIST, while SII can be considered as a prognostic factor. GNRI and SII can be used as tools to evaluate the prognosis of patients before surgery, helping doctors to better treat high-risk patients.

## Introduction

Gastrointestinal mesenchymal tumor (GIST) is the most common type of sarcoma with a low incidence in the population (1). However, with improvements in immunohistochemical analysis and imaging techniques such as endoscopy and ultrasound, there has been a significant increase in the reported incidence of GIST over the past 20 years (2, 3). For example, GISTs have been found in many specimens of patients undergoing bariatric surgery (4). It is reported that more than 12 per million population are currently diagnosed with GIST each year and accounting for only 0.2% of all gastrointestinal tract tumors (5). It is now believed that GIST mainly occurs from Interstitial cells of Cajal (ICC), the cells that trigger gastrointestinal motility, most commonly due to mutations in the receptor tyrosine kinase, especially in adults with mutations in the *KIT* or *PDGFRA* genes (6–8). These mutations lead to constitutive activation of tyrosine kinases and are the basis for targeted molecular therapies. Tyrosine kinase inhibitors (TKIs) are the current first-line clinical drugs (9), but the side effects such as nausea, rash, diarrhea, muscle spasm, fatigue, and periorbital edema during long-term use (10), and the high cost of the drug and the emergence of drug resistance are also reasons that patients cannot continue to use TKIs. The current recommended treatment for GIST is complete surgical resection, but postoperative recurrence is the main cause of reduced survival (11). Therefore, it is very important to identify the risk of recurrence after GIST.

Accurate evaluation of the prognosis of GIST in clinical work is conducive to the choice of subsequent treatment for patients (12). Currently, the most widely used to predict GIST prognosis are the National Institutes of Health (NIH)–Fletcher staging system, Modified NIH (mNIH), Armed Forces Institute of Pathology (AFIP) risk classification, and the American Joint Commission on Cancer (AJCC) staging system (13–16), tumor-specific parameters such as location, mitotic index, and size were included to stratify the risk of recurrence in GIST patients. Although those tumor staging systems for GIST have been developed and validated, results regarding the consistency of each model with recurrence-free survival(RFS) remain controversial. Considering the importance of estimating the risk of recurrence when selecting patients suitable for adjuvant TKI therapy, it is important to identify a risk stratification system with greater predictive power.

Recent studies have shown that inflammatory metrics such as neutrophil-lymphocyte ratio (NLR), lymphocyte-monocyte ratio (LMR), and platelet-lymphocyte ratio (PLR) are significantly correlated with RFS in GIST disease and other cancers (17–19). However, these prognostic factors are usually based on two immune-inflammatory cell types and therefore they have limited predictive reliability for clinical outcomes. Systemic Immunoinflammatory Index (SII), as a composite index based on peripheral blood platelet, neutrophil, and lymphocyte counts, has been extensively studied and has proven to be a valid predictor of prognosis in various malignancies (20–23). Nøst et al. (24) found that compared with NLR, PLR, LMR, and other indicators, SII had the strongest association with the risk of colorectal cancer and lung cancer, and the degree of association increased in the cases diagnosed within one year after recruitment. Katayama et al. (25) found that in bladder cancer, preoperative SII can identify non-muscle invasive bladder cancer patients who have worse disease and prognosis. Keit et al. (26) also found that high SII was associated with low disease control and survival rate of stage III non-small cell lung cancer. However, whether it is associated with recurrent metastasis in GIST has not been reported in a large sample of studies.

In cancer patients, especially those with digestive tract tumors, are prone to malnutrition (27). Many studies have shown that with the occurrence of malnutrition, the postoperative complications and mortality of cancer patients may increase (28). Previous articles have shown that nutritional indicators such as prognosis nutritional index(PNI) is the factor influencing GIST prognosis (29, 30). Bouillanne et al. (31) reported the Geriatric Nutrition Risk Index (GNRI), which is a new and objective nutritional assessment method, with a good effect in predicting the mortality and incidence rate of malnourished elderly people. Wang et al. (32) compared the four nutritional risk indexes Prognostic Nutritional Index (PNI), Nutritional Risk Index (NRI), Controlling Nutritional Status (CONUT) score, and GNRI to predict the prognosis of esophageal cancer. The results showed that GNRI was the most powerful predictor of the four nutritional risk indexes.

Therefore, in this study, our goal was to assess whether common inflammatory and nutritional indicators are associated with RFS in our single-center patients undergoing surgical resection of GIST.

## Methods

### Patients

All patients who underwent radical resection of GIST between October 2004 and July 2018 were inpatients from the Department of General Surgery at Beijing Hospital. Inclusion criteria included (1) Complete surgical resection (R0 resection); (2) Eastern Cooperative Oncology Group (ECOG) performance status score < 3; (3) age ≥18 years; (4) The postoperative survival was more than 1 month; and (5) no adjuvant TKI therapy. Exclusion criteria included (1) history with other primary tumors, (2) incomplete clinical records, (3) history with hematologic disease or infection at the time of blood work; Our study was approved by the institutional research ethics committee.

### Data Collection

Clinical data were obtained retrospectively from the medical record system and included patient age, sex, height, weight, tumor site, tumor size, and mitotic index [number of mitoses per 50 high-powered fields of view (HPF)]. Peripheral blood test data included lymphocyte, neutrophil, serum albumin, monocyte, and platelet count were also collected within 1 week before surgery. At the time of blood collection, no patient had clinical signs of infection. GNRI was calculated as GNRI = [1.489* albumin (g/L)] + [41.7* (actual body weight/ideal body weight)] and ideal weight was calculated from the Lorentz equations (WLo) (31), and when weight > ideal weight, the ratio was treated as 1, SII is defined as Platelet count × Neutrophil count/Lymphocyte count, NLR is defined as neutrophil-lymphocyte ratio, LMR is defined as lymphocyte-monocyte ratio, PNI is defined as 10×serum albumin value (g/dL)+0.005×peripheral lymphocyte count, BMI is defined as height/weight/weight.

### Follow Up

Patients were followed up annually after 5 years of surgery, every 6-12 months for 3-5 years, and every 3-6 months for 2 years. The last follow-up was performed in December 2019. Recurrence-free survival (RFS) was set as the primary endpoint, defined as the time from surgery to tumor recurrence of metastasis or death. Follow-up tasks included routine peripheral blood tests, abdominal ultrasound. Magnetic resonance imaging (MRI) or computed tomography (CT) was used to judge whether there is recurrence or metastasis.

### Statistical Analysis

Categorical variables were expressed in numbers and percentages. Continuous variables with normal and without normal distribution were demonstrated as mean (standard deviation[SD]) and median(interquartile range[IQR]), respectively. Categorical variables were compared by the chi-square test, and continuous variables were compared by Student t-test or Mann-Whitney U test (depending on the normal distribution or not). P-value <0.05 noted statistical significance. Baseline patient characteristics, clinicopathological variables, and outcomes were reported with the R package “**Table 1**”. The optimal cutoff for SII and GNRI differentiation was calculated using x-tile software, which is always used in finding the best cut-off value in survival data (33, 34). The different SII and GNRI subgroups were estimated using the Kaplan-Meier method and compared using the log-rank test. R-package “survivalROC” was used to access the accuracy of the cutoff, the higher the area under the ROC curve (AUC) value represents the higher the prediction accuracy. The Kaplan-Meier method and the log-rank test were used to plot survival curves and compared with the R package “survminer”. Univariate and multivariate analysis was performed using the Cox proportional risk model to determine independent prognostic factors for RFS with the R package “survival”. Confidence intervals (CI) were calculated at the 95% level. All statistical analyses were performed using R software.

**Table 1:** Relationship between clinical characteristics and GNRI AND SII.

level	Overall (n = 160)	GNRI-high (n = 107)	GNRI-low (n = 53)	p	SII-low (n = 139)	SII-high (n = 21)	p
Gender (%)				0.112			0.082
Men	90 (56.2)	55 (51.4)	35 (66.0)		74 (53.2)	16 (76.2)	
Women	70 (43.8)	52 (48.6)	18 (34.0)		65 (46.8)	5 (23.8)	
Age, years (mean (SD))	60.11 (12.06)	59.61 (11.30)	61.11 (13.51)	0.459	59.19 (12.02)	66.14 (10.73)	**0.013**
Tumor size (median [IQR])	4.50 [2.50, 6.73]	3.50 [2.00, 5.50]	5.50 [3.70, 8.50]	**<0.001**	4.00 [2.45, 6.00]	8.00 [4.50, 13.00]	**<0.001**
GNRI (%)				**<0.001**			0.078
≥98.3	107 (66.9)	107 (100.0)	0 (0.0)		97 (69.8)	10 (47.6)	
<98.3	53 (33.1)	0 (0.0)	53 (100.0)		42 (30.2)	11 (52.4)	
SII (%)				0.078			<0.001
<820	139 (86.9)	97 (90.7)	42 (79.2)		139 (100.0)	0 (0.0)	
≥820	21 (13.1)	10 (9.3)	11 (20.8)		0 (0.0)	21 (100.0)	
KI67 (%)				**0.001**			**0.013**
<0.1	143 (89.4)	103 (96.3)	40 (75.5)		128 (92.1)	15 (71.4)	
>0.1	17 (10.6)	4 (3.7)	13 (24.5)		11 (7.9)	6 (28.6)	
Ecog score (%)				0.217			0.394
0	142 (88.8)	98 (91.6)	44 (83.0)		125 (89.9)	17 (81.0)	
1	15 (9.4)	8 (7.5)	7 (13.2)		12 (8.6)	3 (14.3)	
2	3 (1.9)	1 (0.9)	2 (3.8)		2 (1.4)	1 (4.8)	
Surgery				**0.002**			0.184
Laparoscopy	89 (55.6)	50 (46.7)	39 (73.6)		74 (53.2)	15 (71.4)	
Open	71 (44.4)	57 (53.3)	14 (26.4)		65 (46.8)	6 (28.6)	
Rupture (%)				0.719			1
Non-rupture	159 (99.4)	107 (100.0)	52 (98.1)		138 (99.3)	21 (100.0)	
Rupture	1 (0.6)	0 (0.0)	1 (1.9)		1 (0.7)	0 (0.0)	
Tumor location (%)				0.401			0.073
Stomach (%)	114 (71.2)	79 (73.8)	35 (66.0)		103 (74.1)	11 (52.4)	
Other site	46 (28.7)	28 (26.2)	18 (34.0)		36 (25.9)	10 (47.6)	
Mitotic index (per 50HPF)				**<0.001**			0.076
<5	115 (71.9)	85 (79.4)	30 (56.6)		104 (74.8)	11 (52.4)	
5~10	22 (13.8)	15 (14.0)	7 (13.2)		18 (12.9)	4 (19.0)	
>10	23 (14.4)	7 (6.5)	16 (30.2)		17 (12.2)	6 (28.6)	
NIH risk category				**0.008**			**0.007**
Very low (%)	32 (20.0)	28 (26.2)	4 (7.5)		31 (22.3)	1 (4.8)	
Low (%)	53 (33.1)	38 (35.5)	15 (28.3)		50 (36.0)	3 (14.3)	
Intermediate (%)	27 (16.9)	15 (14.0)	12 (22.6)		22 (15.8)	5 (23.8)	
High (%)	48 (30.0)	26 (24.3)	22 (41.5)		36 (25.9)	12 (57.1)	
BMI (mean (SD))	24.08 (3.12)	24.81 (2.75)	22.61 (3.32)	**<0.001**	24.18 (3.19)	23.43 (2.51)	0.301
Rfsmonth (median [IQR])	34.50 [15.00, 67.25]	37.00 [18.00, 68.00]	26.00 [14.00, 61.00]	0.357	38.00 [20.00, 69.50]	12.00 [8.00, 35.00]	**0.002**

SII-low: SII index < 820; SII-high: SII index ≥ 820; GNRI-high: GNRI index ≥ 98.3; GNRI-low: GNRI index < 98.3. The p value of bold is less than 0.05, which is significant.

## Results

### Patient Summary

Based on the strict inclusion and exclusion criteria above, we finally retrospectively analyzed 160 patients’ information, the clinicopathological characteristics are shown in **Table 1**. They had a mean age of 60 ± 12 years for the whole population at the time of diagnosis, of which 56.2% were men. The gastric stromal tumor is the most common stromal tumor (71.2%), with only 28.7% at other sites. The majority of patients (71.9%) had a mitotic rate of five or fewer mitoses per 50 high-powered fields of view (HPF). The rupture occurred in only 1 of our 160 patients, and the median tumor size was 4.5 cm [2.50, 6.73]. Almost 32 (20.0%), 53 (33.1%), 27 (16.9%), and 48 (30.0%) patients were classified into very low, low, intermediate, and high-risk groups according to the mNIH GIST risk classification.

### Comparison of Common Indicators and Identification of Optimal Median Points

**Figure 1:**
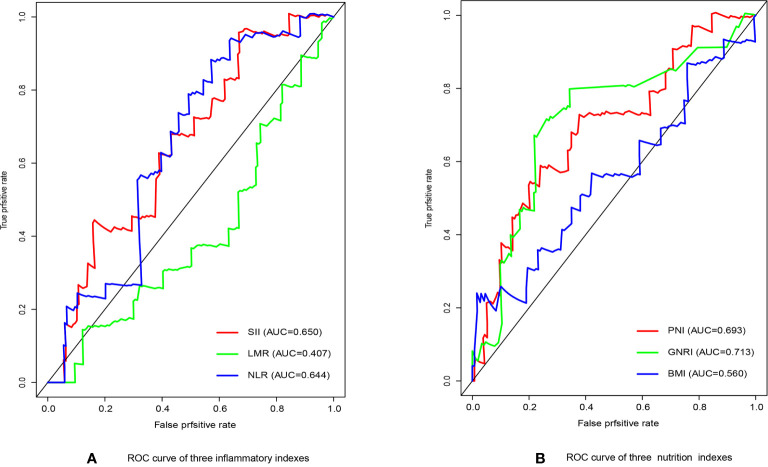
**(A)** The receiver operating characteristic (ROC) analysis of inflammatory indicators. **(B)** The receiver operating characteristic (ROC) analysis of nutritional indicators.

**Figure 2:**
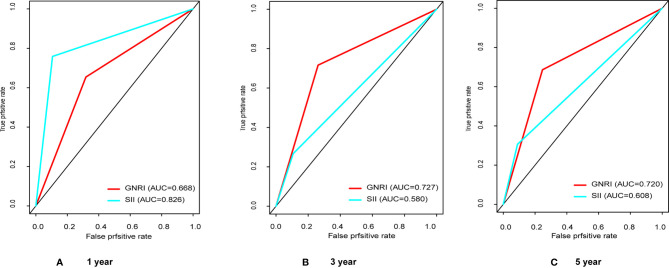
The receiver operating characteristic (ROC) analysis of GNRI and SII. **(A)** The areas under the curve (AUC) in 1 year for GNRI and SII were 0.668 and 0.826; **(B)** The areas under the curve (AUC) in 3 years for GNRI and SII were 0.727 and 0.580; **(C)** The areas under the curve (AUC) in 5 years for GNRI and SII were 0.720 and 0.608.

We have performed ROC curve analysis for commonly used inflammatory and nutritional indicators in order to compare with these indexes, and the area under the curve (AUC) represents the accuracy of predicting prognosis. The results are shown in **Figure 1**, where the AUC of SII and GNRI are the largest, reaching 0.650 and 0.713 respectively. The ranges of GNRI, and SII were 75.9-113.2 and 116-3481, respectively. To assess the optimal cut-off values, we used X-tile to assess the optimal cut-off values for RFS, and the results are shown in **Supplementary Figure 1**, where their optimal cutoff was 98.3, and 820.0, respectively. We also made ROC curves at this cutoff, as shown in **Figure 2**, the AUC for GNRI and SII reached 0.668 and 0.826 at 1 year; the 3-year AUC was 0.727 and 0.580, and the 5-year AUC was 0.720 and 0.608, respectively, showing their high prognostic predictive power.

### Correlation of GNRI and SII With Clinicopathological Features

To verify the relationship between GNRI and SII and clinicopathological features, we performed a correlation analysis, and the results are displayed in **Table 1**. The gender and age were no significant differences between the high and low GNRI groups (p = 0.112, p= 0.459). However, tumor size, mitotic index, proliferation index ki67, BMI, surgery method, and NIH risk category are all correlated with GNRI classification (all p < 0.05). SII did not correlate with gender (p = 0.082), but correlated with age, the older patients with the higher inflammation (p = 0.013), while proliferation index, size and NIH classification were also correlated to SII (p < 0.001, p = 0.013, p=0.007), Median survival was also significantly higher in patients with low systemic inflammatory index (p = 0.002).

### Survival Analysis and Subgroup Analysis

**Figure 3:**
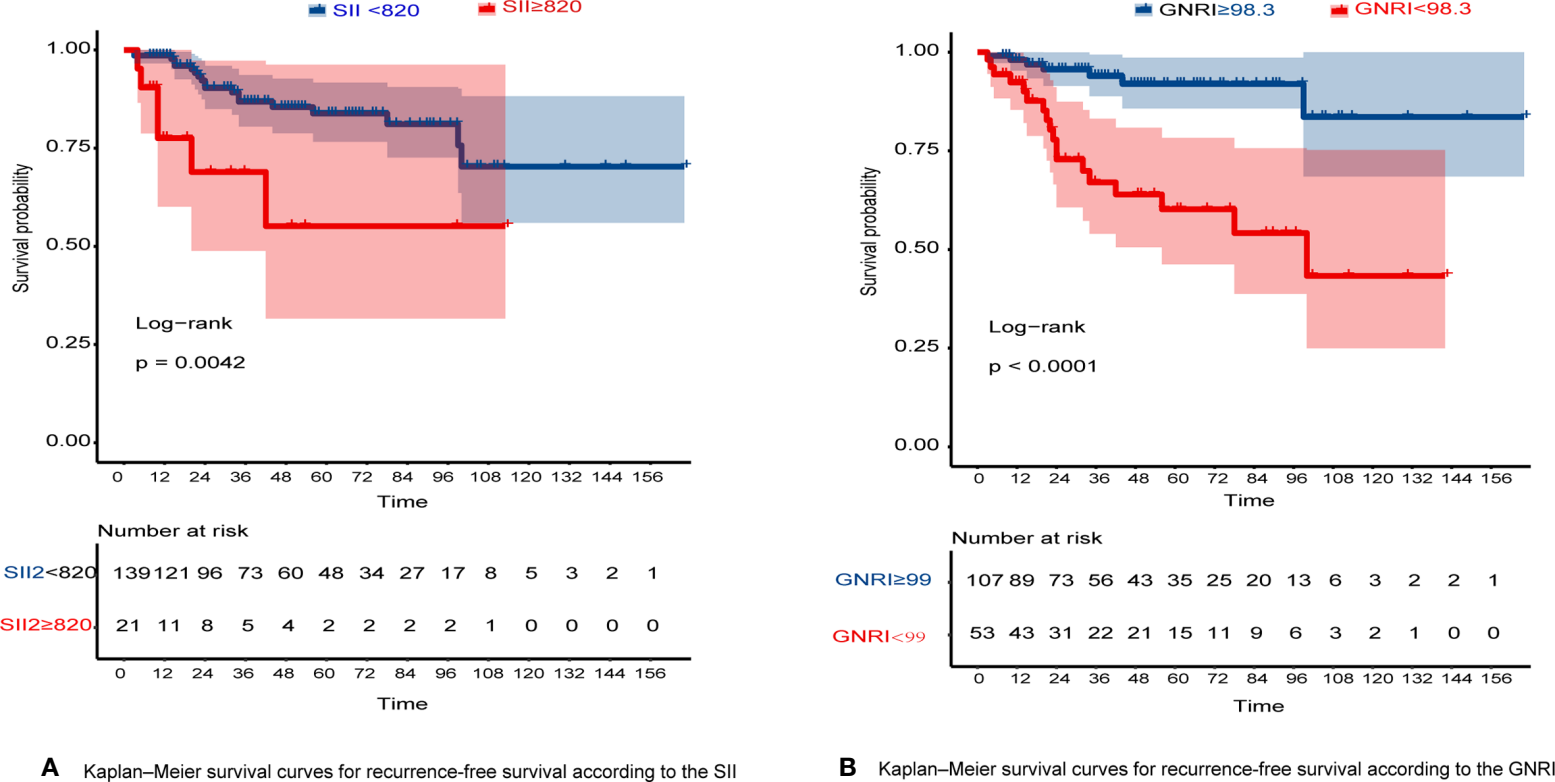
**(A)** Kaplan–Meier survival curves for RFS according to the SII; **(B)** Kaplan–Meier survival curves for RFS according to the GNRI.

**Figure 4:**
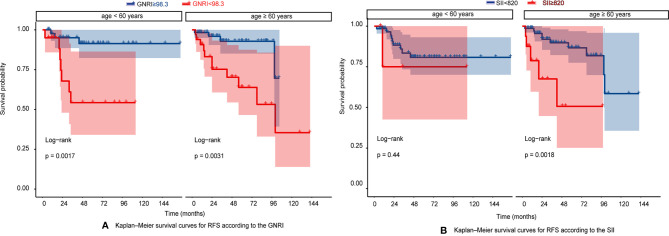
**(A)** Kaplan–Meier survival curves for RFS according to the GNRI in very age <60 years and age > 60 years; **(B)** Kaplan–Meier survival curves for RFS according to the SII in very age <60 years and age > 60 years.

**Figure 5:**
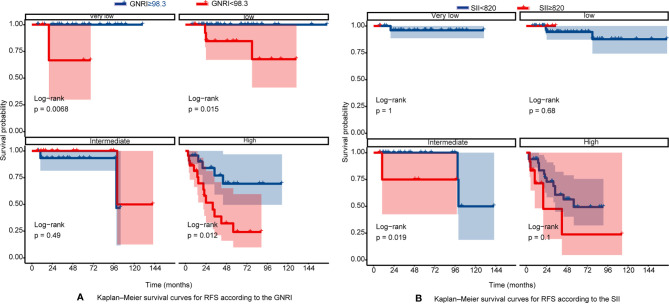
**(A)** Kaplan–Meier survival curves for RFS according to the GNRI in different mNIH stage; **(B)** Kaplan–Meier survival curves for RFS according to the SII in different mNIH stage.

The RFS rates of GIST were 96.1%, 84.7%, and 80.8% at years 1, 3, and 5, respectively. Among all patients, 10 patients (6.25%) experienced tumor recurrence and metastasis. Patients in the high GNRI group’s 3-year and 5-year RFS rates were 93.95% and 91.96%, while 66.99% and 60.19% in the low GNRI group, as shown in **Figure 3A**. In the SII analysis, the overall RFS was also all different, Patients in the low SII group had significantly higher RFS than those in the high SII group (3-year RFS rate of 86.82% *versus* 68.90% and 5-year RFS rate of 83.82% *versus* 55.10%, as shown in **Figure 3B**). We also performed subgroup analysis in young and old people, and the results showed in **Figure 4** that GNRI not only distinguishes between high- and low-risk patients in older patients (p=0.003) but also in younger patients (p=0.001). But in different SII classes, only the elderly had a significant difference (p=0.001), there was no significant difference in the young (p=0.44). In addition, we performed a subgroup analysis of the different mNIH classifications to determine the ability to differentiate at different stages and the results are shown in **Figure 5**, for GNRI there was no significance at the intermediate stage (p=0.49) but significant at the low and high-risk stages (p<0.05). However, for SII there was significance in the intermediate stage (p=0.019) and a higher prognosis for low inflammatory index, but not in the other stages (p>0.05).

### Univariate and Multivariate Analyses

**Figure 6:**
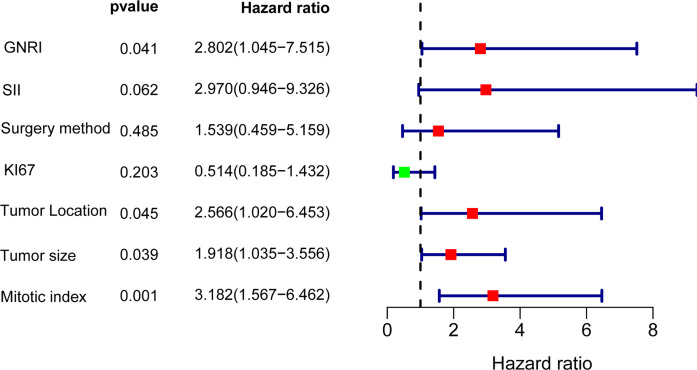
The forest plot of hazard ratio after multivariate Cox regression analysis.

To further analyze the factors affecting the RFS, we performed univariate and multifactorial cox analyses, and the results are presented in **Table 2**. In the univariate analysis, sex, age, and Ecog score were not risk factors (p>0.05), the other factor such as GNRI, SII, KI67, surgery method, tumor site, tumor size, and mitotic index are remarkable predictors of RFS. Incorporation of the above indicators into multivariate analysis revealed GNRI (HR=2.802,95% CI: 1.045 to 7.515, p = 0.041), tumor site (HR=2.566,95% CI: 1.020 to 6.453, p = 0.045), mitotic index (HR=3.182,95% CI: 1.567 to 6.462, p = 0.001) and tumor size (HR = 1.918, 95% CI: 1.035 to 3.556, p = 0.039) were independent prognostic factors for GIST, in addition SII also tended to be associated with prognosis (HR = 2.970, 95% CI: 0.946 to 9.326, p = 0.062), the results of the multifactorial analysis are also shown in Forest (**Figure 6**).

**Table 2:** Univariate and multivariate analysis of the prognostic factors for recurrence-free survival in patients with GIST.

Variables	Univariate analysis				Multivariate analysis			
	HR	HR.95Low	HR.95High	p	HR	HR.95Low	HR.95High	p
Sex	0.80	0.35	1.82	0.601				
Age	1.60	0.73	3.50	0.245				
GNRI	5.64	2.36	13.52	<0.001	2.80	1.05	7.52	**0.041**
SII	3.56	1.41	8.99	0.007	2.97	0.95	9.33	0.062
KI67	7.37	3.29	16.51	<0.001	0.51	0.19	1.43	0.203
Ecog	1.15	0.41	3.20	0.794				
Surgical method	0.31	0.12	0.83	0.020	1.54	0.46	5.16	0.485
Tumor site	4.62	2.08	10.25	<0.001	2.57	1.02	6.45	**0.045**
Tumor size	3.45	2.16	5.52	<0.001	1.92	1.04	3.56	**0.039**
Mitotic index	3.94	2.44	6.37	<0.001	3.18	1.57	6.46	**0.001**

HR, hazard ratio. The p value of bold is less than 0.05, which is significant.

## Discussion

GIST is the most common sarcoma, and the most common and effective treatment is surgical resection with negative margins, but recurrence and metastasis of the original disease after surgery are important reasons for the shortened survival time. Therefore, it is particularly important to predict the risk of recurrence and metastasis, thus assisting in the early clinical screening of patients with a poor prognosis for targeted therapy.

Chronic inflammation has been previously reported to be associated with long-term prognosis in various cancers (35). Previous studies have shown that peripheral biomarkers of blood PLR and NLR have been correlated with recurrence-free survival and overall survival after gastrointestinal mesenchymal tumor surgery (36, 37). They have also been shown to have independent prognostic utility for a variety of cancers, including esophageal, ovarian, pancreatic, colorectal, and endometrial malignancies (38–40). However, the above metrics are all ratios of two blood indicators, and SII has recently been found to be more accurate in predicting survival in many cancers (41, 42), but no large-sample studies have yet examined the relationship between SII and GIST outcomes. We retrospectively analyzed the data and we found that RFS was significantly better in the low SII group, 3- and 5-year RFS rates for patients with low SII (≤ 820) were 86.82% and 83.82% compared with 68.90% and 55.10% for patients with higher SII (> 820). And in the univariate analysis showed that it was a risk factor for RFS, while correcting for multiple factors showed that it was not significant (the HR = 2.970, 95% CI: 0.946 to 9.326, p = 0.062), which we believe is also suggestive for prognosis. To further test their ability for prognosis, we tested their ROC curves, and the area under the curve reached 0.608 in 5-year recurrence-free survival, representing its good predictive function for recurrence-free survival. However, in subgroup analysis, the performance of SII was a little poor, only in the elderly and patients with intermediate stage, showing a good ability to distinguish.

As a composite index based on peripheral blood platelet, neutrophil, and lymphocyte count, patients with elevated SII count usually present with elevated inflammatory status and a weakened immune response. The prediction for tumor recurrence and metastasis can be elucidated by their relationship with circulating tumor cells (CTCs). Elevated platelets can interact with tumor cells through different mechanisms to promote tumor cell epithelial-mesenchymal transition for metastasis (43). Neutrophils have been reported to have the ability to enhance cancer cell invasion, proliferation, and metastasis by establishing a tumor microenvironment (44). Peripheral lymphocytes are significantly correlated with tumor-infiltrating lymphocytes(TIL). When the circulating lymphocytes decrease, the infiltrating lymphocytes also decrease, so the ability to fight cancer cells decreases, leading to a poor prognosis (45). Activated lymphocytes play a fundamental role in cell-mediated immune destruction of host cancer cells (46). All of these may lead to more tumor cells spreading into the circulatory system, evading immune surveillance, and ultimately increasing peripheral CTC levels.

Furthermore, it is well known that malnutrition will increase the incidence of postoperative complications, which harms long-term survival. Timely nutritional support can reduce the incidence of postoperative complications and hospitalization rates (47). GNRI is a modification of the Nutritional Risk Index (NRI) because NRI requires current weight divided by previous weight, but since many elderly or other patients do not weigh themselves often or forget their previous weight, the authors further corrected NRI by calculating ideal weight instead of previous weight, so GNRI is more accurate and convenient in practice. GNRI serves as a valid predictor which has been reported to be a prognostic factor of many cancers, such as pancreatic cancer (48), advanced non-small cell lung cancer (49), large B cell lymphoma (50), gastric carcinomas (51), and so on. But there is no relevant research in GIST. We were the first to demonstrate that GNRI was the risk factor for patients with GIST. The optional cutoff value is 98.3, which is similar to the cutoff values in other cancers and studies (52). We found that RFS was significantly better in the high GNRI group, 3- and 5-year RFS rates for patients with high GNRI (>98.3) were 93.95% and 91.96% compared to 66.99% and 60.19% for patients with low GNRI group. The GNRI was initially set up as a model formula primarily to include the elderly population because it is difficult to remember their previous weight for calculation. For many young people, they also face the situation that their previous weight is not very accurate. Using the ratio of current weight to ideal weight is more reflective of the specific nutritional status of the current patient, so does GNRI apply to young people as well? Related studies have shown its applicability to younger patients as well (53, 54). In this study, we further conducted subgroup analysis in the young and the elderly, as showed in **Figure 4**, the survival curves of the two groups were both significant. The performance is also great at different mNIH stages, especially in high-risk stage, the prognosis of patients with high GNRI is significantly better, so doctors can target therapy or expand surgery for high-risk patients with low GNRI.

GNRI was composed of serum albumin level and body weight ratio [actual body weight (ABW)/ideal body weight (IBW)], and the ratio of ABW and IBW can more objectively reflect the weight changes in oncology patients due to tumor depletion. Hypoalbuminemia has been proved to be a harmful consequence of many malignant tumors. The decrease of albumin reduces the body’s ability to cope with variable stresses, such as malignant tumors and surgery (55). GIST can lead to a variety of digestive symptoms, as intestinal obstruction and gastrointestinal bleeding, constipation, and diarrhea (56). These factors may contribute to impaired nutritional status. In this study, the bigger tumor, higher KI67, and higher mitotic index related to the low GNRI, which proves that the growth of the tumor consumes more energy, leading to malnutrition and poor prognosis. Therefore, GNRI can be considered a convenient and useful biomarker for assessing the preoperative prognosis of GIST patients. Furthermore, the results in the multifactorial analysis showed that tumor size, tumor site, and mitotic index (per 50HPF) were also risk factors, and this obtained conclusion is consistent with the results of existing guidelines.

This study also has several limitations. Firstly, previous studies have shown that tumor rupture is also an important prognostic factor, but in the present study, only one patient presented with tumor rupture, which may cause too much bias when including multiple factors and therefore was not included in the multifactorial analysis. In addition, patients with GIST who received adjuvant TKI therapy were excluded. Therefore, it may be a kind of selection bias while excluding some patients with intermediate or high-risk tumors. Then, this was a single-institution retrospective study. Ultimately, further larger samples and studies are needed to demonstrate the independent association between peripheral biomarkers SII, GNRI, and long-term oncologic outcomes.

## Conclusion

In conclusion, GNRI and SII are simple and useful markers to predict the RFS after surgical resection of gastrointestinal stromal tumors. To our knowledge, this is the first large-scale study to demonstrate the prognostic importance of SII and GNRI in patients with GIST, especially GNRI. We believe that these indicators can not only help determine whether to proceed with adjuvant therapy but also help determine the scope and method of the surgery.

## Data Availability Statement

The raw data supporting the conclusions of this article will be made available by the authors, without undue reservation.

## Ethics Statement

The studies involving human participants were reviewed and approved by Ethics Committee of Beijing hospital. Written informed consent for participation was not required for this study in accordance with the national legislation and the institutional requirements.

## Author Contributions

ZL and RL were involved in the final data analysis and manuscript writing. XC, CL, ZS, XS, WS, and YZ were assisted in the data collection and analysis. JS was involved in study design and responsible for the entire research project. All authors have agreed on the journal to which the article has been submitted, agree to be accountable for all aspects of the work criteria, made a significant contribution to the work reported. All authors contributed to the article and approved the submitted version.

## Conflict of Interest

The authors declare that the research was conducted in the absence of any commercial or financial relationships that could be construed as a potential conflict of interest.

## Publisher’s Note

All claims expressed in this article are solely those of the authors and do not necessarily represent those of their affiliated organizations, or those of the publisher, the editors and the reviewers. Any product that may be evaluated in this article, or claim that may be made by its manufacturer, is not guaranteed or endorsed by the publisher.

## References

[1] Joensuu H. Gastrointestinal Stromal Tumor (GIST). Ann Oncol (2006) 17:x280–6. 10.1093/annonc/mdl274 17018739

[2] Hirota S. Differential Diagnosis of Gastrointestinal Stromal Tumor by Histopathology and Immunohistochemistry. Trans Gastroenterol Hepatol (2018) 3:27. 10.21037/tgh.2018.04.01 PMC6002266 29971258

[3] Ahmed M. Recent Advances in the Management of Gastrointestinal Stromal Tumor. World J Clin cases (2020) 8(15):3142. 10.12998/wjcc.v8.i15.3142 32874969 PMC7441252

[4] Lyros O Moulla Y Mehdorn M Schierle K Sucher R Dietrich A. Coincidental Detection of Gastrointestinal Stromal Tumors During Laparoscopic Bariatric Procedures-Data and Treatment Strategy of a German Reference Center. Obes Surg (2019) 29(6):1858–66. 10.1007/s11695-019-03782-y 30875013

[5] Blay JY Kang YK Nishida T von Mehren M. Gastrointestinal Stromal Tumours. Nat Rev Dis Primers (2021) 7(1):22. 10.1038/s41572-021-00254-5 33737510

[6] Taniguchi M Nishida T Hirota S Isozaki K Ito T Nomura T. Effect of C-Kit Mutation on Prognosis of Gastrointestinal Stromal Tumors. Cancer Res (1999) 59(17):4297–300. 10485475

[7] Rubin BP Singer S Tsao C Duensing A Lux ML Ruiz R. KIT Activation is a Ubiquitous Feature of Gastrointestinal Stromal Tumors. Cancer Res (2001) 61(22):8118–21. 11719439

[8] Roland CL Feig BW. History of GIST. Gastrointestinal Stromal Tumors. Springer (2017). p:1–5.

[9] Lim KT Tan KY. Current Research and Treatment for Gastrointestinal Stromal Tumors. World J Gastroenterol (2017) 23(27):4856. 10.3748/wjg.v23.i27.4856 28785140 PMC5526756

[10] Zhang Q Xu J Qian Y Chen L Li Q Xu K. Association of Imatinib Plasma Concentration and Single-Nucleotide Polymorphisms With Adverse Drug Reactions in Patients With Gastrointestinal Stromal Tumors. Mol Cancer Ther (2018) 17(12):2780–7. 10.1158/1535-7163.MCT-18-0498 30282814

[11] Gatto L Nannini M Saponara M Di Scioscio V Beltramo G Frezza GP. Radiotherapy in the Management of Gist: State of the Art and New Potential Scenarios. Clin Sarcoma Res (2017) 7(1):1–6. 10.1186/s13569-016-0065-z 28078078 PMC5223331

[12] Supsamutchai C Wilasrusmee C Hiranyatheb P Jirasiritham J Rakchob T Choikrua P. A Cohort Study of Prognostic Factors Associated With Recurrence or Metastasis of Gastrointestinal Stromal Tumor (GIST) of Stomach. Ann Med Surg (2018) 35:1–5. 10.1016/j.amsu.2018.08.010 PMC6153116 30258625

[13] Fletcher CD Berman JJ Corless C Gorstein F Lasota J Longley BJ. Diagnosis of Gastrointestinal Stromal Tumors: A Consensus Approach. Hum Pathol (2002) 33(5):459–65. 10.1053/hupa.2002.123545 12094370

[14] Miettinen M Lasota J. Gastrointestinal Stromal Tumors: Review on Morphology, Molecular Pathology, Prognosis, and Differential Diagnosis. Arch Pathol Lab Med (2006) 130(10):1466–78. 10.5858/2006-130-1466-GSTROM 17090188

[15] Joensuu H. Risk Stratification of Patients Diagnosed With Gastrointestinal Stromal Tumor. Hum Pathol (2008) 39(10):1411–9. 10.1016/j.humpath.2008.06.025 18774375

[16] Chen T Xu L Ye L Qiu H Hu Y Liu H. A New Nomogram for Recurrence-Free Survival Prediction of Gastrointestinal Stromal Tumors: Comparison With Current Risk Classification Methods. Eur J Surg Oncol (2019) 45(6):1109–14. 10.1016/j.ejso.2018.12.014 30594406

[17] Goh BK Chok AY Allen JC Jr Quek R Teo MC Chow PK. Blood Neutrophil-to-Lymphocyte and Platelet-to-Lymphocyte Ratios Are Independent Prognostic Factors for Surgically Resected Gastrointestinal Stromal Tumors. Surgery (2016) 159(4):1146–56. 10.1016/j.surg.2015.10.021 26688506

[18] Stotz M Liegl-Atzwanger B Posch F Mrsic E Thalhammer M Stojakovic T. Blood-Based Biomarkers Are Associated With Disease Recurrence and Survival in Gastrointestinal Stroma Tumor Patients After Surgical Resection. PloS One (2016) 11(7):e0159448. 10.1371/journal.pone.0159448 27454486 PMC4959723

[19] Romano L Mattei A Colozzi S Giuliani A Cianca G Lazzarin G. Laparoscopic Sleeve Gastrectomy: A Role of Inflammatory Markers in the Early Detection of Gastric Leak. J Minim Access Surg (2020). 10.4103/jmas.JMAS_3_20 PMC8270050 32964887

[20] Hu B Yang XR Xu Y Sun YF Sun C Guo W. Systemic Immune-Inflammation Index Predicts Prognosis of Patients After Curative Resection for Hepatocellular Carcinoma. Clin Cancer Res Off J Am Assoc Cancer Res (2014) 20(23):6212–22. 10.1158/1078-0432.Ccr-14-0442 25271081

[21] Murthy P Zenati MS Al Abbas AI Rieser CJ Bahary N Lotze MT. Prognostic Value of the Systemic Immune-Inflammation Index (SII) After Neoadjuvant Therapy for Patients With Resected Pancreatic Cancer. Ann Surg Oncol (2020) 27(3):898–906. 10.1245/s10434-019-08094-0 31792715 PMC7879583

[22] Hua X Long Z-Q Zhang Y-L Wen W Guo L Xia W. Prognostic Value of Preoperative Systemic Immune-Inflammation Index in Breast Cancer: A Propensity Score-Matching Study. Front Oncol (2020) 10:580. 10.3389/fonc.2020.00580 32373539 PMC7186330

[23] Wang D Hu X Xiao L Long G Yao L Wang Z. Prognostic Nutritional Index and Systemic Immune-Inflammation Index Predict the Prognosis of Patients With HCC. J Gastrointestinal Surg (2020) 25(2):421–7. 10.1007/s11605-019-04492-7 PMC7904713 32026332

[24] Nøst TH Alcala K Urbarova I Byrne KS Guida F Sandanger TM. Systemic Inflammation Markers and Cancer Incidence in the UK Biobank. Eur J Epidemiol (2021). 10.1007/s10654-021-00752-6 PMC8416852 34036468

[25] Katayama S Mori K Pradere B Laukhtina E Schuettfort VM Quhal F. Prognostic Value of the Systemic Immune-Inflammation Index in Non-Muscle Invasive Bladder Cancer. World J Urol (2021). 10.1007/s00345-021-03740-3 PMC8602174 34143284

[26] Keit E Coutu B Zhen W Zhang C Lin C Bennion N. Systemic Inflammation Is Associated With Inferior Disease Control and Survival in Stage III Non-Small Cell Lung Cancer. Ann Transl Med (2021) 9(3):227. 10.21037/atm-20-6710 33708854 PMC7940875

[27] Van Cutsem E Arends J. The Causes and Consequences of Cancer-Associated Malnutrition. Eur J Oncol Nurs (2005) 9:S51–63. 10.1016/j.ejon.2005.09.007 16437758

[28] Planas M Álvarez-Hernández J León-Sanz M Celaya-Pérez S Araujo K De Lorenzo AG. Prevalence of Hospital Malnutrition in Cancer Patients: A Sub-Analysis of the PREDyCES® Study. Support Care Cancer (2016) 24(1):429–35. 10.1007/s00520-015-2813-7 26099900

[29] Sun J Mei Y Zhu Q Shou C Tjhoi WEH Yang W. Relationship of Prognostic Nutritional Index With Prognosis of Gastrointestinal Stromal Tumors. J Cancer (2019) 10(12):2679. 10.7150/jca.32299 31258776 PMC6584938

[30] Bao X Liu F Lin J Chen Q Chen L Chen F. Nutritional Assessment and Prognosis of Oral Cancer Patients: A Large-Scale Prospective Study. BMC Cancer (2020) 20(1):1–8. 10.1186/s12885-020-6604-2 PMC7036168 32087695

[31] Bouillanne O Morineau G Dupont C Coulombel I Vincent J-P Nicolis I. Geriatric Nutritional Risk Index: A New Index for Evaluating at-Risk Elderly Medical Patients. Am J Clin Nutr (2005) 82(4):777–83. 10.1093/ajcn/82.4.777 16210706

[32] Wang PY Chen XK Liu Q Xu L Zhang RX Liu XB. Application of Four Nutritional Risk Indexes in Perioperative Management for Esophageal Cancer Patients. J Cancer Res Clin Oncol (2021) 1–13. 10.1007/s00432-021-03585-8 33687565 PMC7941130

[33] Zhao Y Deng Y Peng J Sui Q Lin J Qiu M. Does the Preoperative Prognostic Nutritional Index Predict Survival in Patients With Liver Metastases From Colorectal Cancer Who Underwent Curative Resection? J Cancer (2018) 9(12):2167. 10.7150/jca.25346 29937936 PMC6010682

[34] Sun Y Zhang Y Huang Z Chi P. Prognostic Implication of Negative Lymph Node Count in Ypn+ Rectal Cancer After Neoadjuvant Chemoradiotherapy and Construction of a Prediction Nomogram. J Gastrointestinal Surg (2019) 23(5):1006–14. 10.1007/s11605-018-3942-3 30187336

[35] Deans C Wigmore SJ. Systemic Inflammation, Cachexia and Prognosis in Patients With Cancer. Curr Opin Clin Nutr Metab Care (2005) 8(3):265–9. 10.1097/01.mco.0000165004.93707.88 15809528

[36] Perez DR Baser RE Cavnar MJ Balachandran VP Antonescu CR Tap WD. Blood Neutrophil-to-Lymphocyte Ratio Is Prognostic in Gastrointestinal Stromal Tumor. Ann Surg Oncol (2013) 20(2):593–9. 10.1245/s10434-012-2682-y PMC3700425 23054118

[37] Feng F Tian Y Liu S Zheng G Liu Z Xu G. Combination of PLR, MLR, MWR, and Tumor Size Could Significantly Increase the Prognostic Value for Gastrointestinal Stromal Tumors. Medicine (2016) 95(14)e3248. 10.1097/MD.0000000000003248 PMC4998783 27057867

[38] Ergen ŞA Barlas C Yıldırım C Öksüz DÇ. Prognostic Role of Peripheral Neutrophil-Lymphocyte Ratio (NLR) and Platelet-Lymphocyte Ratio (PLR) in Patients With Rectal Cancer Undergoing Neoadjuvant Chemoradiotherapy. J Gastrointestinal Cancer (2021) 1–10. 10.1007/s12029-020-00578-7 33392960

[39] Zhai Z Gao J Zhu Z Cong X Lou S Han B. The Ratio of the Hemoglobin to Red Cell Distribution Width Combined With the Ratio of Platelets to Lymphocytes Can Predict the Survival of Patients With Gastric Cancer Liver Metastasis. BioMed Res Int (2021) 2021. 10.1155/2021/8729869 PMC7814956 33506035

[40] Watanabe J Otani S Sakamoto T Arai Y Hanaki T Amisaki M. Prognostic Indicators Based on Inflammatory and Nutritional Factors After Pancreaticoduodenectomy for Pancreatic Cancer. Surg Today (2016) 46(11):1258–67. 10.1007/s00595-016-1308-6 26869184

[41] Hu L Huang X You C Li J Hong K Li P. Prevalence of Overweight, Obesity, Abdominal Obesity and Obesity-Related Risk Factors in Southern China. PloS One (2017) 12(9). 10.1371/journal.pone.0183934 PMC5598943 28910301

[42] Yang R Chang Q Meng X Gao N Wang W. Prognostic Value of Systemic Immune-Inflammation Index in Cancer: A Meta-Analysis. J Cancer (2018) 9(18):3295. 10.7150/jca.25691 30271489 PMC6160683

[43] Labelle M Begum S Hynes RO. Direct Signaling Between Platelets and Cancer Cells Induces an Epithelial-Mesenchymal-Like Transition and Promotes Metastasis. Cancer Cell (2011) 20(5):576–90. 10.1016/j.ccr.2011.09.009 PMC3487108 22094253

[44] Dumitru CA Lang S Brandau S. eds. Modulation of Neutrophil Granulocytes in the Tumor Microenvironment: Mechanisms and Consequences for Tumor Progression. Elsevier (2013). 10.1016/j.semcancer.2013.02.005 23485549

[45] Lee KH Kim EY Yun JS Park YL Do SI Chae SW. The Prognostic and Predictive Value of Tumor-Infiltrating Lymphocytes and Hematologic Parameters in Patients With Breast Cancer. BMC Cancer (2018) 18(1):938. 10.1186/s12885-018-4832-5 30285668 PMC6167816

[46] Reading JL Gálvez-Cancino F Swanton C Lladser A Peggs KS Quezada SA. The Function and Dysfunction of Memory CD8(+) T Cells in Tumor Immunity. Immunological Rev (2018) 283(1):194–212. 10.1111/imr.12657 29664561

[47] Baracos VE. Cancer-Associated Malnutrition. Eur J Clin Nutr (2018) 72(9):1255–9. 10.1038/s41430-018-0245-4 30185853

[48] Sakamoto T Yagyu T Uchinaka E Miyatani K Hanaki T Kihara K. The Prognostic Significance of Combined Geriatric Nutritional Risk Index and Psoas Muscle Volume in Older Patients With Pancreatic Cancer. BMC Cancer (2021) 21(1):342. 10.1186/s12885-021-08094-y 33789590 PMC8011217

[49] Sonehara K Tateishi K Araki T Komatsu M Yamamoto H Hanaoka M. Prognostic Value of the Geriatric Nutritional Risk Index Among Patients With Previously Treated Advanced Non-Small Cell Lung Cancer Who Subsequently Underwent Immunotherapy. Thorac Cancer (2021) 12(9):1366–72. 10.1111/1759-7714.13909 PMC8088948 33710780

[50] Matsukawa T Suto K Kanaya M Izumiyama K Minauchi K Yoshida S. Validation and Comparison of Prognostic Values of GNRI, PNI, and CONUT in Newly Diagnosed Diffuse Large B Cell Lymphoma. Ann Hematol (2020) 99(12):2859–68. 10.1007/s00277-020-04262-5 32970194

[51] Sugawara K Yamashita H Urabe M Okumura Y Yagi K Aikou S. Geriatric Nutrition Index Influences Survival Outcomes in Gastric Carcinoma Patients Undergoing Radical Surgery. JPEN J Parenter Enteral Nutr (2020). 10.1002/jpen.1978 32740962

[52] Xie H Tang S Wei L Gan J. Geriatric Nutritional Risk Index as a Predictor of Complications and Long-Term Outcomes in Patients With Gastrointestinal Malignancy: A Systematic Review and Meta-Analysis. Cancer Cell Int (2020) 20(1):530. 10.1186/s12935-020-01628-7 33292289 PMC7603782

[53] Lidoriki I Schizas D Frountzas M Machairas N Prodromidou A Kapelouzou A. GNRI as a Prognostic Factor for Outcomes in Cancer Patients: A Systematic Review of the Literature. Nutr Cancer (2021) 73(3):391–403. 10.1080/01635581.2020.1756350 32321298

[54] Lee GW Go SI Kim DW Kim HG Kim JH An HJ. Geriatric Nutritional Risk Index as a Prognostic Marker in Patients With Extensive-Stage Disease Small Cell Lung Cancer: Results From a Randomized Controlled Trial. Thorac Cancer (2020) 11(1):62–71. 10.1111/1759-7714.13229 31707767 PMC6938749

[55] Hoogenboezem EN Duvall CL. Harnessing Albumin as a Carrier for Cancer Therapies. Adv Drug Deliv Rev (2018) 130:73–89. 10.1016/j.addr.2018.07.011 30012492 PMC6200408

[56] Caterino S Lorenzon L Petrucciani N Iannicelli E Pilozzi E Romiti A. Gastrointestinal Stromal Tumors: Correlation Between Symptoms at Presentation, Tumor Location and Prognostic Factors in 47 Consecutive Patients. World J Surg Oncol (2011) 9(1):1–10. 10.1186/1477-7819-9-13 21284869 PMC3039617

