# Supplementary material for: Assessment of Systemic Inflammation and Nutritional Indicators in Predicting Recurrence-Free Survival After Surgical Resection of Gastrointestinal Stromal Tumors
Source: Front Oncol. 2021 Jul 26;11:710191. doi: 10.3389/fonc.2021.710191 (PMC8350728; doi:10.3389/fonc.2021.710191)
Supplement: Supplementary file 1 [file DataSheet_1.docx]

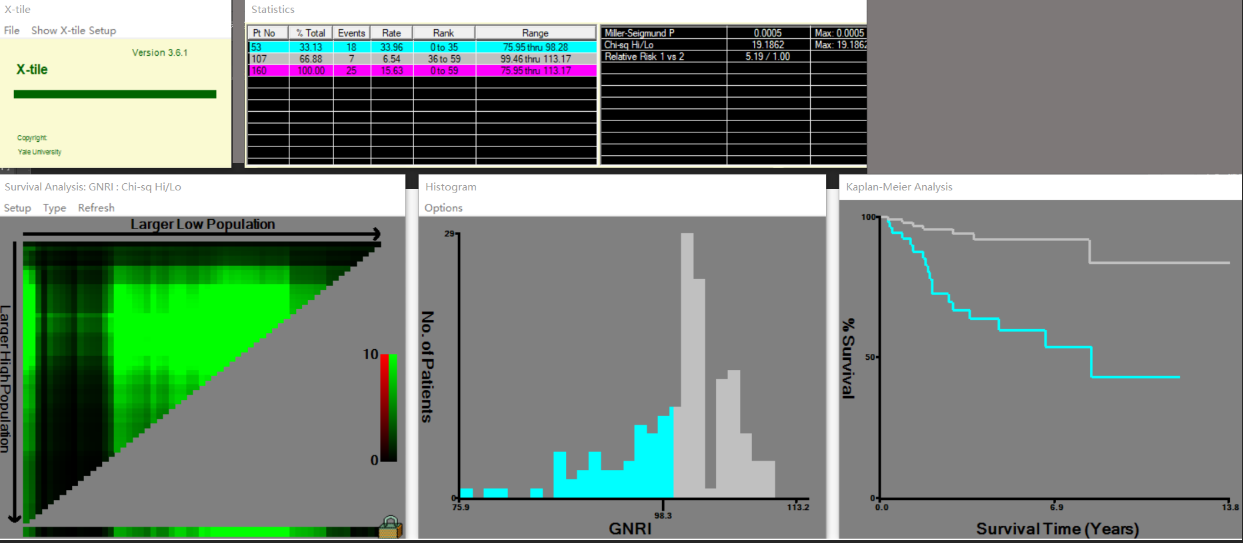


Supplementary Figure 1. Optimal cutoff of GNRI was 98.3 by X-tile.


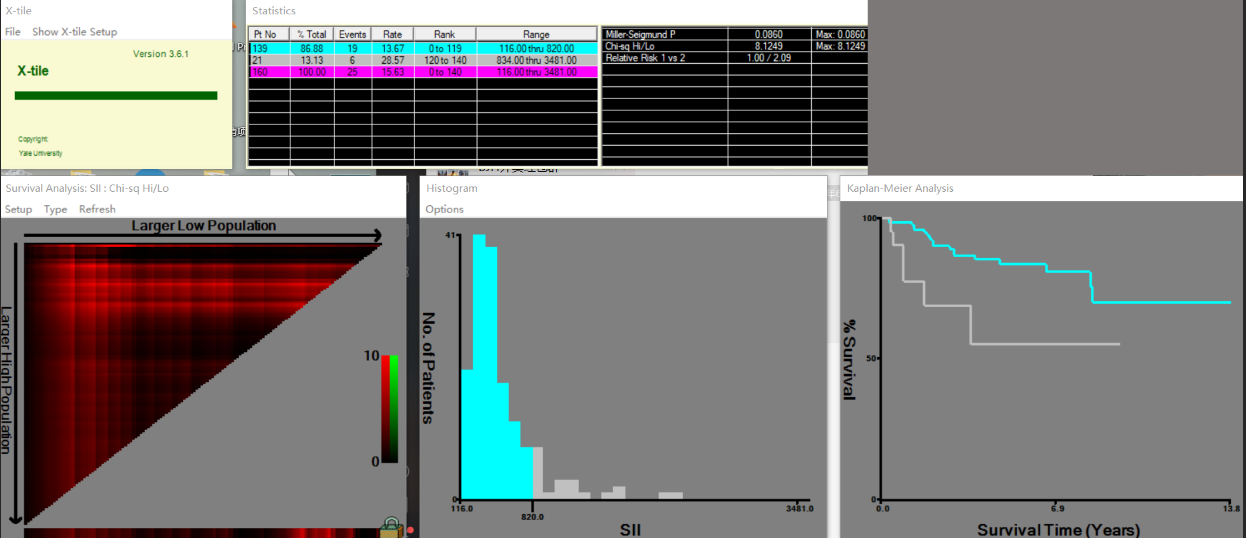


Supplementary Figure 2. Optimal cutoff of sii was 820.0 by X-tile.
